# Creation of a new type of ion exchange material for rapid, high-capacity, reversible and selective ion exchange without swelling and entrainment†
†Electronic supplementary information (ESI) available. See DOI: 10.1039/c5sc04507j


**DOI:** 10.1039/c5sc04507j

**Published:** 2015-12-14

**Authors:** Baiyan Li, Yiming Zhang, Dingxuan Ma, Zhenyu Xing, Tianliang Ma, Zhan Shi, Xiulei Ji, Shengqian Ma

**Affiliations:** a Department of Chemistry , University of South Florida , 4202 E. Fowler Avenue , Tampa , FL 33620 , USA . Email: sqma@usf.edu; b State Key Laboratory of Inorganic Synthesis and Preparative Chemistry , College of Chemistry , Jilin University , Changchun 130012 , People's Republic of China . Email: zshi@jlu.edu.cn; c Department of Chemistry , Oregon State University , 2100 SW Monroe Ave. , Corvallis , OR , USA 97331; d College of Resource and Environmental Science , Jilin Agricultural University , Changchun 130118 , China

## Abstract

Ion-exchange materials, currently dominated by resins, are widely used in a plethora of areas. However, the drawbacks of conventional resins necessitate the creation of a new model of ion exchange materials that feature controllable swelling, easily accessible ion exchange sites, high ion exchange capacity, fast ion exchange kinetics, and high chemical stability as illustrated herein in the context of functionalizing a porous organic polymer (POP) with ion exchange groups. The advantages of POP-based ion exchange materials in comparison with conventional resins and other types of ion exchange materials have been highlighted through an evaluation of their performances in scavenging precious metals at trace concentrations, removal of nuclear waste model ions, and size-selective ion capture. Our work thereby provides a new perspective to develop ion functionalized POPs as a versatile type of ion exchange materials for various applications.

## Abstract 2

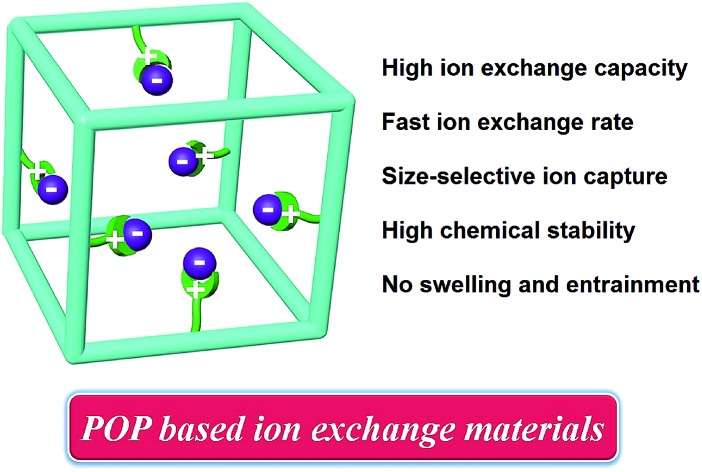
A new model for ion exchange materials has been proposed on the basis of ion exchange sites grafted to a porous organic polymer.

## Introduction

Ion-exchange materials play an important role in areas including water treatment,^1^ ion pollutant removal,^2^ and ion separation.^3^ Molecular sieves and ceramic materials, although well explored as ion-exchange materials, exhibit a slow ion-exchange behavior and low capacity, thus limiting their wide use in practical applications.^4^ Metal–organic frameworks (MOFs)^5^–^7^ also present themselves as a new generation of ion-exchange materials, with work already showing potential applications in anionic pollutant removal^8^–^12^ and selective anion exchange.^13^–^16^ But the instabilities associated with the majority of MOFs, particularly under harsh conditions (*i.e.* strong acid/base), largely limit their real practical application as ion-exchange materials. In addition, a majority of MOFs are hydrophilic frameworks, which is not of benefit for fast ion exchange kinetics in aqueous solution. To date, ion-exchange resins dominate the applied ion exchange field.^17^ However, conventional ion exchange resins often face several unsolved drawbacks including uncontrolled swelling as well as inefficient accessibility of the ion-exchange sites to ions as a result of the flexible feature of polymer chains and uneven distribution and entrainment of the charged sites,^17^ which therefore would lead to a decrease of mechanical strength, low ion exchange capacity, limited kinetics and “outflow” of mobile phase under working conditions.^18^ The strategy of employing MOF^4^ materials and ordered mesoporous silica^19^ as hosts with the goal of confining the ion-exchange polymer chains has been developed to prevent the swelling and entanglement. But these composite materials often suffer from drawbacks such as significant diffusion resistance, low ion exchange capacity due to the extra weight of the host framework, and chemical instability of the host materials under a wide pH range (pH = 0–14). The weaknesses of existing ion-exchange materials necessitate the development of new robust alternatives that control swelling, provide readily accessible ion exchange sites, and possess a high ion exchange capacity and fast ion exchange kinetics.

To overcome the aforementioned challenges, we propose a new model of ion exchange materials with the following features: (1) a rigid framework to prevent swelling; (2) a monolayer open pore wall to avoid entrainment; (3) a hydrophobic backbone to enhance the ion mobility in aqueous solution thus resulting in fast ion exchange kinetics; (4) a high density of ion exchange sites contributing to a high ion exchange capacity; (5) a strong irreversible covalent bond in order to obtain a high chemical stability.

**Scheme 1:**
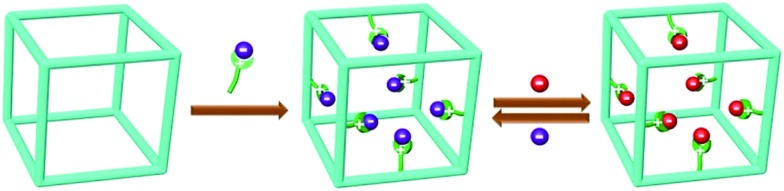
Illustration of functionalizing POP for reversible anion exchange.

Such a new model of ion exchange materials can be realised *via* functionalizing porous organic polymers (POPs),^20^–^24^ which have recently been developed as a new type of porous materials because of their amenability of design and modular nature, high surface areas, adjustable pore sizes, functionalizable surfaces, and exceptional chemical stability as well as their potential for applications in areas including gas storage/separation,^25^–^38^ catalysis,^39^–^45^ pollutant removal,^46^–^48^ and energy storage.^49^–^54^ We speculate that if ion-exchange groups can be grafted to the hydrophobic backbones of the highly porous robust POP framework, a high density of readily accessible ion-exchange sites that are arranged into a three-dimensional nanospace will be achieved (Scheme 1). This is anticipated to afford new ion exchange materials with a high ion-exchange capacity, fast ion-exchange kinetics, together with controlled swelling, easily accessible ion exchange sites as well as a high chemical stability. In addition, the well tailorable framework and controllable pore size of POPs provide an opportunity to tune the framework to selectively adsorb ion guest molecules *via* size-exclusion,^15^ a property conventional ion-exchange resins fail to provide.

In this contribution, we demonstrate, for the first time, a new type of ion exchange material capable of rapidly exchanging ions with a high capacity, great reversibility and extra high chemical stability without swelling or entrainment. The afforded POP-based ion exchange materials can be used as a versatile platform in the ion exchange-based separation process. To the best of our knowledge, the material presented is the only kind of ion exchange material possessing all of the features given above (Table 1). Our studies therefore not only lay a foundation for developing POPs as a new type of ion exchange material circumventing the issues of swelling and entanglement encountered in conventional ion exchange resins, but also advance POP-based ion exchange materials as a new platform for applications in ion selective separation and purification.

**Table 1:** Comparison of the features of POP-based ion exchange materials with other types of ion exchange materials

Ion materials	Stability^*a*^	Ion exchange rate	Ion exchange capacity	Swelling	Ion sites entanglement
POP-based ion exchange material	Yes	Fast	High	No	No
Ion-exchange resins	Yes	Fast	High	Yes	Yes
Resin composite with MOF or mesoporous silica	No	Fast	Low	No	No
Molecular sieves	Yes	Slow	Low	No	No
Ceramic materials	Yes	Slow	Low	No	No
MOF	No	Slow	High	No	No

^*a*^In both strong acid and strong base.

## Results and discussion

### Synthesis and characterization

**Scheme 2:**
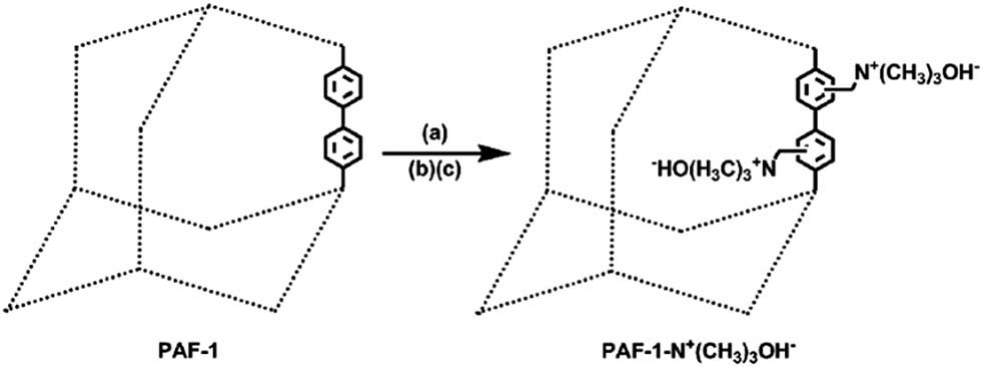
Synthetic route of PAF-1-CH_2_N^+^(CH_3_)_3_OH^–^. (a) CH_3_COOH/HCl/H_3_PO_4_/HCHO, 363 K, 3 days; (b) trimethylamine, ethanol, 353 K, 3 days; (c) 1 M NaOH, twice.

We chose PAF-1 (ref. 55) (PAF = porous aromatic framework) [also known as (a.k.a.) PPN-6]^56^,^57^ as the model material for the “proof of concept” because of its very high surface area and exceptional water/chemical stabilities. In principle, a desired ion-exchange group can be grafted onto any POPs for either cation exchange or anion exchange. Herein, we focus on functionalizing the POP for anion exchange as exemplified by grafting the strong basic trimethylammonium hydroxide moiety onto PAF-1. Chloromethylation of PAF-1 followed by the treatment with trimethylamine in ethanol yielded PAF-1-CH_2_N^+^(CH_3_)_3_Cl^–^. PAF-1-CH_2_N^+^(CH_3_)_3_OH^–^ was obtained *via* ion exchange of PAF-1-CH_2_N^+^(CH_3_)_3_Cl^–^ in 1 M NaOH (Scheme 2, Fig. S1 and S2, ESI†).

**Fig. 1:**
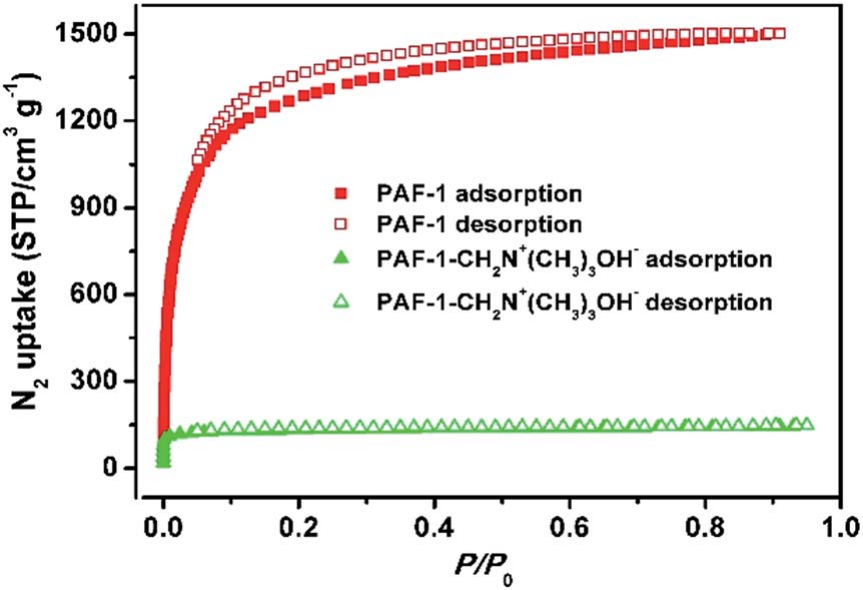
N_2_ sorption isotherms of PAF-1 (red) and PAF-1-CH_2_N^+^(CH_3_)_3_OH^–^ (green).

The successful grafting of the trimethylammonium hydroxide moiety onto PAF-1 was confirmed using Fourier transform infrared spectroscopy (FT-IR), solid-state ^13^C NMR and elemental analysis studies. When compared with pristine PAF-1, the FT-IR spectra of dehydrated PAF-1-CH_2_N^+^(CH_3_)_3_OH^–^ shows the aliphatic C–H stretching band at 2953 cm^–1^ and the characteristic band for C–N at 1280 cm^–1^ (Fig. S3, ESI†). Solid-state ^13^C NMR studies show the chemical shifts of CH_3_ and CH_2_ at 51.9 ppm and 63.0 ppm, suggesting the successful grafting of –CH_2_N^+^(CH_3_)_3_OH^–^ groups to the phenyl rings in PAF-1 (Fig. S4, ESI†). Elemental analysis reveals a nitrogen content of 3.88 wt% corresponding to 2.8 mmol g^–1^ of CH_2_N^+^(CH_3_)_3_OH^–^ groups in PAF-1-CH_2_N^+^(CH_3_)_3_OH^–^, suggesting 44% of the phenyl rings are grafted with one CH_2_N^+^(CH_3_)_3_OH^–^ group. N_2_ sorption isotherms collected at 77 K (Fig. 1) show a significant decrease in the Brunauer–Emmett–Teller (BET) surface area from 4715 to 505 m^2^ g^–1^ and a reduction of pore volume from 2.0 to 0.27 cm^3^ g^–1^ after modification of PAF-1 with –CH_2_N^+^(CH_3_)_3_OH^–^. Meanwhile, the pore size is also reduced from ∼1.5 nm for PAF-1 to ∼1.1 nm for PAF-1-CH_2_N^+^(CH_3_)_3_OH^–^ (Fig. S5, ESI†). These results are consistent with the modification of the functional groups onto the POPs.

### Ion exchange kinetic performances of POP-based ion exchange materials

**Fig. 2:**
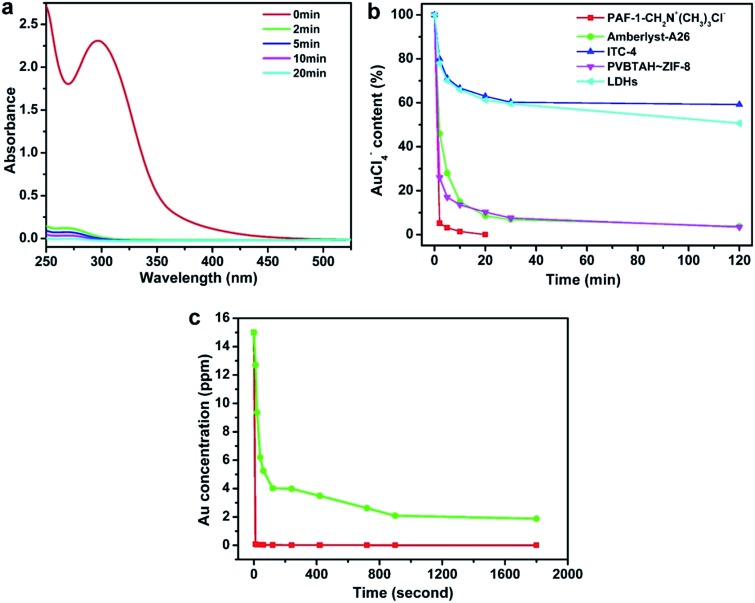
Kinetics investigation of PAF-1-CH_2_N^+^(CH_3_)_3_Cl^–^. (a) UV-vis spectra of AuCl_4_^–^ aqueous solution in the presence of PAF-1-CH_2_N^+^(CH_3_)_3_Cl^–^ monitored with time. (b) Comparison of the ion exchange performances of PAF-1-CH_2_N^+^(CH_3_)_3_Cl^–^ and other ion exchange materials in extracting AuCl_4_^–^. (c) Au(CN)_2_^–^ exchange kinetics of PAF-1-CH_2_N^+^(CH_3_)_3_OH^–^ (red) and Amberlyst-A26 (green) with an Au(i) initial concentration of 15 ppm in KAu(CN)_2_ solution.

The extraction of AuX_4_^–^ (X = Cl or Br) or Au(CN)_2_^–^ are two different routes for scavenging precious metal from precious metal electroplating waste water, which features economic and environmental incentives.^4^,^58^ To evaluate the merit of the POP based ion exchange materials, we used the extraction of the AuCl_4_^–^ ion from aqueous solutions as a model experiment. We observed PAF-1-CH_2_N^+^(CH_3_)_3_Cl^–^ extracting 96% of the AuCl_4_^–^ ion within 2 min (Fig. 2a). The commercial ion exchange resin, Amberlyst-A26 possessing the same ion-exchange group N^+^(CH_3_)_3_Cl^–^ and the composited ion exchange material PVBTAH–ZIF-8^4^ took at least 30 min to extract the same amount of the AuCl_4_^–^ ion under the same conditions (Fig. 2b, S6, ESI†). We also tested the ion capture performances of LDHs,^59^ ITC-4,^15^ ZIF-8,^60^ and PAF-1 using the same AuCl_4_^–^ model experiment (Fig. 2b, S6, ESI†). These materials exhibit a low ion capture ability and slow kinetic behaviors extracting ∼25–50% of the AuCl_4_^–^ ion after 120 min. We also compared the ion exchange performances of stable ion exchange materials under such conditions including PAF-1-CH_2_N^+^(CH_3_)_3_Cl^–^, Amberlyst-A26 and LDHs based on the same amount of active exchange sites, and PAF-1-CH_2_N^+^(CH_3_)_3_Cl^–^ also shows significant advantages over Amberlyst-A26 and LDHs (Fig. S7, ESI†). Furthermore, we examined the performances of PAF-1-CH_2_N^+^(CH_3_)_3_OH^–^ as an ion-exchange material in extracting ppm levels of gold cyanide in water solution. As shown in Fig. 2c, PAF-1-CH_2_N^+^(CH_3_)_3_OH^–^ can rapidly capture Au(CN)_2_^–^ ions; and >99% of the Au(CN)_2_^–^ anion can be extracted within 10 seconds, which is in striking contrast with only 15% extraction of the Au(CN)_2_^–^ anion by the Amberlyst-A26 commercial resin. Equilibrium adsorption is also established at 10 seconds for PAF-1-CH_2_N^+^(CH_3_)_3_OH^–^, compared to 15 min for Amberlyst-A26. A similar trend was also observed when using PAF-1-CH_2_N^+^(CH_3_)_3_OH^–^ and Amberlyst-A26 with the same amount of ion exchange sites (Fig. S8, ESI†). The appearance of the IR peak at 2144 cm^–1^ can be attributed to the uptake of Au(CN)_2_^–^ by PAF-1-CH_2_N^+^(CH_3_)_3_OH^–^ (Fig. S9, ESI†). The adsorption rate constant (*k*_2_) was fitted with the pseudo-second-order kinetic model (Fig. S10 and S11, ESI†) and the value was determined to be 50.4 g mg^–1^ min^–1^ (141 mmol mg^–1^ min^–1^) for PAF-1-CH_2_N^+^(CH_3_)_3_OH^–^, which is two order-of-magnitude higher than the Amberlyst-A26 resin with a *k*_2_ value of 0.25 g mg^–1^ min^–1^ (0.78 mmol mg^–1^ min^–1^) under the same conditions. The fast ion exchange of the POP-based ion exchange materials can be attributed to the highly accessible ion-exchange sites in the open pores and the fast ion mobility in aqueous solution benefiting from the framework hydrophobicity as well as the strong coulombic interactions between the charged framework and extracts. To gain further insight into the mobility behavior of ions in the porous framework, we measured the conductivity of Au(CN)_2_^–^@PAF-1-CH_2_N^+^(CH_3_)_3_OH^–^ and Au(CN)_2_^–^@Amberlyst-A26 (Fig. S12, ESI†). Analysis of Au(CN)_2_^–^@PAF-1-CH_2_N^+^(CH_3_)_3_OH^–^ in an ambient environment gave a conductivity of 3.23 × 10^–7^ S cm^–1^ while the same measurement on Au(CN)_2_^–^@Amberlyst-A26 produced a conductivity of 1.21 × 10^–7^ S cm^–1^, indicating a higher Au(CN)_2_^–^ ion mobility in PAF-1-CH_2_N^+^(CH_3_)_3_OH^–^.^16^ These results are consistent with the faster extraction kinetics observed in PAF-1-CH_2_N^+^(CH_3_)_3_OH^–^.

### Ion exchange capacity of POP-based ion exchange materials

**Fig. 3:**
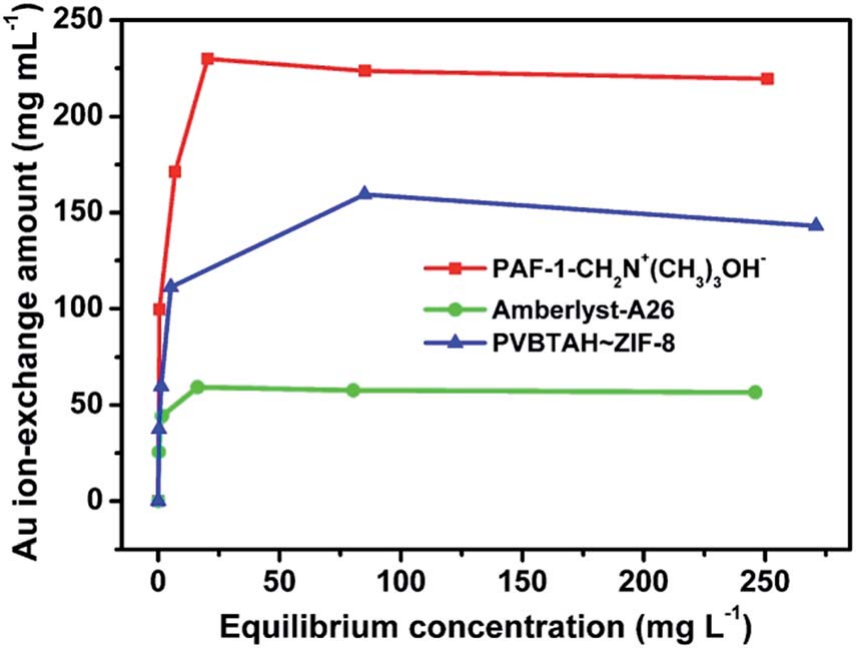
Volumetric Au(i) adsorption isotherms for PAF-1-CH_2_N^+^(CH_3_)_3_OH^–^, Amberlyst-A26 and PVBTAH–ZIF-8.

The dry weight ion-exchange capacity of PAF-1-CH_2_N^+^(CH_3_)_3_Cl^–^ measured by AgNO_3_ titration is 3.4 meq g^–1^, comparable to that of Amberlyst-A26 (Table S1, ESI†). However, the wet volume ion-exchange capacity (2.4 meq mL^–1^) of PAF-1-CH_2_N^+^(CH_3_)_3_OH^–^ is three times that of Amberlyst-A26 (0.8 meq mL^–1^) (Table S1, ESI†), which experiences dramatic swelling as a result of the nature of its flexible polymer chains. We also assessed the maximum working capacities of PAF-1-CH_2_N^+^(CH_3_)_3_OH^–^ and Amberlyst-A26 using Au(CN)_2_^–^ extraction. The equilibrium adsorption isotherm data, fitted using the Langmuir model, yielded a high correlation coefficient (>0.9998) (Fig. S13 and S14, ESI†). Under the same working conditions, the maximum dry weight working capacity of PAF-1-CH_2_N^+^(CH_3_)_3_OH^–^ is comparable to that of Amberlyst-A26 (Fig. S15, ESI†). Nonetheless, the wet volume working capacity of PAF-1-CH_2_N^+^(CH_3_)_3_OH^–^ is 2.9 times higher than that of Amberlyst-A26 (Fig. 3). We ascribe the significant difference to the high density of highly accessible ion-exchange sites distributed on the rigid 3D framework. Furthermore, another reason for the high wet volume working capacity of PAF-1-CH_2_N^+^(CH_3_)_3_OH^–^ should stem from the inherent robust framework of PAF-1,^55^,^56^ which does not exhibit the possible swelling as observed for flexible polymers.^4^,^18^ In addition, both the dry weight ion-exchange capacity and wet volume ion-exchange capacity of PAF-1-CH_2_N^+^(CH_3_)_3_OH^–^ are also higher than the composited ion exchange material PVBTAH–ZIF-8 (Fig. 3, S15 and S16, ESI†). This should presumably be due to the addition of the extra MOF framework, decreasing effective ion exchange sites in the composited materials. In addition, the volumetric uptake amount of Au(CN)_2_^–^ in PAF-1-CH_2_N^+^(CH_3_)_3_OH^–^ is also higher than in Amberlyst-A26 with the same mol of active exchange sites (Fig. S17, ESI†). These results further highlight the advantages of the functionalized POPs as a new platform for ion exchange.

### Investigation of POP-based ion exchange materials in nuclear waste model ion removal

**Fig. 4:**
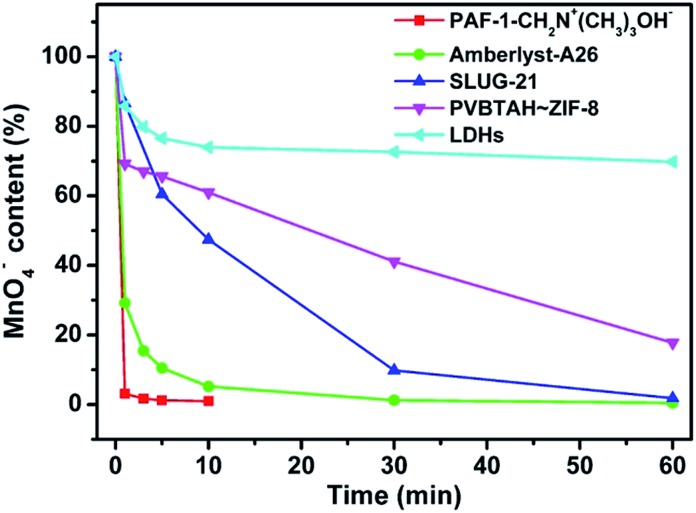
Comparison of the ion exchange performances of PAF-1-CH_2_N^+^(CH_3_)_3_OH^–^ and other ion exchange materials in removing model MnO_4_^–^ ions.

In addition, we also investigated the potential application of POP-based ion exchange materials for the removal of radioactive technetium (Tc-99), which is a highly problematic ion in nuclear waste. Permanganate has been used as the model ion for studying pertechnetate uptake since both are group 7 oxo-anions.^9^ As shown in Fig. 4, almost 99% of the MnO_4_^–^ can be removed by PAF-1-CH_2_N^+^(CH_3_)_3_OH^–^ in less than 5 min, whereas it takes at least 30 min to reach the same removal capacity for commercial Amberlyst-A26 under the same conditions. Furthermore, we also compared the ion exchange performance of PAF-1-CH_2_N^+^(CH_3_)_3_OH^–^ with other anion exchange materials including LDHs, PVBTAH–ZIF-8 and SLUG-21 (ref. 9) (Fig. 4, S18, ESI†). They show an even worse capability in removing the MnO_4_^–^ ions, and even after 60 min only 31%, 92% and 98% of the MnO_4_^–^ ions can be removed for LDHs, PVBTAH–ZIF-8 and SLUG-21, respectively (Fig. 4). For the stable ion exchange materials of PAF-1-CH_2_N^+^(CH_3_)_3_OH^–^, Amberlyst-A26 and LDHs with the same mol of active exchange sites, PAF-1-CH_2_N^+^(CH_3_)_3_OH^–^ is also superior to Amberlyst-A26 and LDHs (Fig. S19, ESI†). These results suggest that POP based ion exchange materials will have obvious advantages for the removal of pertechnetate ions when compared with other types of ion exchange materials.

### Size selective ion exchange in POP-based ion exchange materials

**Fig. 5:**
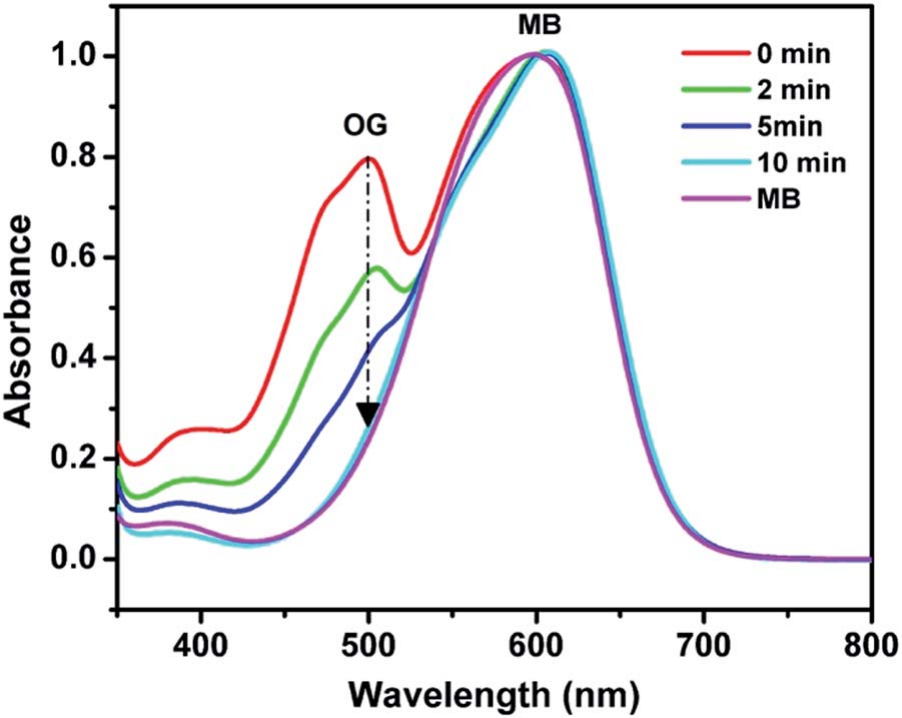
UV-vis spectra of OG/MB aqueous solution in the presence of PAF-1-CH_2_N^+^(CH_3_)_3_OH^–^.

Beyond the fast ion exchange rate, high ion exchange capacity, and controllable swelling, POP-based ion exchange materials can be employed to selectively capture ion compounds *via* a size-exclusion effect. To illustrate the size-selective ion capture, two anionic dyes, Methyl Blue (MB) and Orange G (OG) which have the same charges but different dimensions (13.89 × 14.35 × 24.49 Å for MB *vs.* 5.44 × 10.14 × 15.64 Å for OG) were used for investigations. Given that the molecular dimensions of OG along a certain orientation are smaller than the pore size of PAF-1-CH_2_N^+^(CH_3_)_3_OH^–^ (11–12.7 Å), PAF-1-CH_2_N^+^(CH_3_)_3_OH^–^ can quickly and completely capture the OG molecules in 10 minutes, whereas the MB molecules remain in the solution (Fig. 5). In contrast, conventional resins and LDHs, with accessible charges on the particle surfaces, fail to effectively separate dye molecules *via* a size exclusion effect (Fig. S20a and b, ESI†). In addition, the MOF ITC-4 was also used to separate two dyes in aqueous solution as a control. The larger MB molecules instead of the smaller OG molecules were quickly extracted in ICT-4 (Fig. S20c, ESI†). This could presumably be due to the framework collapse of ICT-4 in an aqueous environment, which remains an issue in practical application for the majority of MOFs.^56^

### Stability study and reversible ion exchange in POP-based ion exchange materials

Considering that harsh conditions such as strong acid and strong base environments are often involved in the application of ion exchange materials, high chemical stability is an essential criterion for an ion exchange material. The high chemical stability of PAF-1-CH_2_N^+^(CH_3_)_3_OH^–^ was verified by immersing the PAF-1-CH_2_N^+^(CH_3_)_3_OH^–^ sample successively with 1.0 M HCl and 1.0 M NaOH. The sample experienced virtually no surface area drop based on the N_2_ sorption isotherms collected at 77 K (Fig. S21, ESI†). This is advantageous compared to mesoporous silica and MOF materials, which experience framework collapse after the same treatments because of their chemical instability under such harsh conditions (Fig. S22 and S23, ESI†).

The fast and reversible ion-exchange of PAF-1-CH_2_N^+^(CH_3_)_3_OH^–^ was examined by soaking the Au(CN)_2_^–^ anion loaded PAF-1-CH_2_N^+^(CH_3_)_3_OH^–^ (Au(CN)_2_^–^ content: 1.03 mmol g^–1^) in 1 M NaOH ethanolic solution (water : ethanol, 1 : 1 v/v). Over 90% of the Au(CN)_2_^–^ anion was eluted in 40 seconds and a complete elution was obtained within 5 min (Fig. S24, ESI†). PAF-1-CH_2_N^+^(CH_3_)_3_OH^–^ can be readily recycled as proven by virtually no loss of Au(i) uptake capacity after five cycles (Fig. S25, ESI†). Nonetheless, for the practical application of ion exchange materials in the recovery of gold, the elution of these ions is mainly on the basis of passing anions contained in aqueous solution through an Au(CN)_2_^–^ accumulated ion exchange column;^61^ this aspect of work will be conducted in the near future.

It is envisioned that the ideal ion-exchange material possesses a high ion exchange capacity (both gravimetric and volumetric), rapid ion exchange rate (fast ion exchange kinetics), high chemical stability (under both strong acidic and basic conditions), and ease of regeneration as well as negligible swelling and minimum entrainment. This could be targeted *via* functionalizing a highly porous and highly robust porous organic polymer (POP) with ion exchange groups as exemplified herein in the context of grafting the strong basic trimethylammonium hydroxide moiety onto the POP of PAF-1 to afford PAF-1-CH_2_N^+^(CH_3_)_3_OH^–^ for anion exchange, which outperforms the benchmark resin of Amberlyst-A26 and other types of ion-exchange materials. In principle, outstanding performances in cation exchange can also be anticipated when a desired cation exchange site is grafted into POPs, and work along this line is currently underway in our laboratory. Although the high cost of PAF-1 would be a concern for the practical utilization of functionalized PAF-1 for ion exchange, the ion exchange groups can be readily grafted into other POPs that are constructed from various organic building blocks derived from a variety of resources through economical reaction processes,^19^,^46^ thus paving a way to develop functionalized POPs as a new type of ion-exchange material for rapid, high-capacity, reversible and selective ion exchange without swelling and entrainment.

## Conclusions

In summary, we have proposed a new model of ion exchange materials that feature highly open pores, monolayer pore walls, and a covalently linked rigid hydrophobic framework *via* grafting ion exchange sites onto porous organic polymers (POPs). The resultant POP-based ion exchange materials exhibit a high ion exchange capacity, fast ion exchange kinetics, and high chemical stability, and meanwhile can overcome the drawbacks of other ion-exchange materials, particularly swelling and entanglement for conventional ion exchange resins, as exemplified in the studies on scavenging precious metals at trace concentrations and removal of nuclear waste model ions. In addition, POP-based ion exchange materials can be designed for the selective capture of ions, a property conventional ion exchange resin cannot provide. Our results highlight the advantages of POP-based ion exchange materials compared to other types of ion exchange materials, and thereby advance POP-based ion exchange materials as a new platform for applications in ion selective separation and purification.

## Experimental

### Material synthesis

A re-sealable flask was charged with PAF-1 (200.0 mg), paraformaldehyde (1.0 g), glacial AcOH (6.0 mL), H_3_PO_4_ (3.0 mL), and conc. HCl (20.0 mL). The flask was sealed and heated to 90 °C for 3 days. The resulting solid was collected, washed with water and methanol, and then dried under vacuum to produce a yellow solid of PAF-1-CH_2_Cl.^57^ Subsequently the obtained PAF-1-CH_2_Cl was mixed with 33% trimethylamine ethanol (3.0 g) in 100 mL of EtOH under N_2_ and stirred at 75 °C for 3 days. The resulting solid was collected, washed with water and methanol, and then dried under vacuum to produce PAF-1-CH_2_N^+^(CH_3_)_3_Cl^–^ as a yellow powder. Then the PAF-1-CH_2_N^+^(CH_3_)_3_Cl^–^ was exchanged using 100 mL of NaOH (1 M) twice to afford PAF-1-CH_2_N^+^(CH_3_)_3_OH^–^. Elemental analysis: experimental result: C: 60.44%; H: 7.28%; N: 3.88%; calculated result (based on one functional group per two phenyl cycles): C: 80.16%; H: 7.69%; N: 5.67%.

### Ion-exchange experiments for AuCl_4_^–^

A 40 mL aqueous solution of KAuCl_4_ (0.835 mM) was added to a 40 mL vial, which was followed by the addition of 20.0 mg samples to form a slurry. During the stirring period, the mixture was filtered at intervals through a 0.45 micron membrane filter for all samples, then the filtrates were analyzed using UV-vis to determine the concentration of the AuCl_4_^–^ ions.

### Ion conductivity studies

The maximum Au(CN)_2_^–^ ion loaded samples were used in the conductivity experiment, which were synthesized based on the following: PAF-1-CH_2_N^+^(CH_3_)_3_OH^–^ and Amberlyst-A26 (100.0 mg) were added to each Erlenmeyer flask containing 1000 ppm KAu(CN)_2_ solution (50 mL). The mixtures were stirred at room temperature for 3 h, and then were filtered and washed using water and methanol, then dried under vacuum to obtain Au(CN)_2_^–^@PAF-1-CH_2_N^+^(CH_3_)_3_OH^–^ and Au(CN)_2_^–^@Amberlyst-A26 for further tests. Pellets of compacted powder sample (13 mm in diameter, thickness around 1 mm) were made using the IR pellet at 50 Mpa for 3 minutes. Then the pellets were sandwiched between two gold foils and put into the Swagelok for an AC impedance spectroscopy measurement. The EIS measurement is performed using the Biologic VMP3 with a frequency range between 1 MHz and 1 Hz and a 50 mV (peak voltage) was applied as AC signals.

## Supplementary Material

Supplementary information
