# Supplementary material for: Creation of a new type of ion exchange material for rapid, high-capacity, reversible and selective ion exchange without swelling and entrainment
Source: Chem Sci. 2015 Dec 14;7(3):2138–44. doi: 10.1039/c5sc04507j (PMC5968557; doi:10.1039/c5sc04507j)
Supplement: Supplementary file 1 [file SC-007-C5SC04507J-s001.pdf]

Electronic Supplementary Material (ESI) for Chemical Science.
This journal is © The Royal Society of Chemistry 2015

Electronic Supplementary Information

Creation of a New Type of Ion Exchange Materials for Rapid, High-Capacity, Reversible and Selective Ion Exchange without Swelling and Entrainment

Baiyan Li,[a] Yiming Zhang,[a] Dingxuan Ma,[b] Zhenyu Xing,[c] Tianliang Ma,[d] Zhan Shi,*[b] Xiulei Ji,[c] and Shengqian Ma*[a]

[a]Department of Chemistry, University of South Florida, 4202 E. Fowler Avenue, Tampa, FL 33620 USA.

E-mail: sqma@usf.edu

[b]State Key Laboratory of Inorganic Synthesis and Preparative Chemistry, College of Chemistry, Jilin University, Changchun 130012, People's Republic of China.

E-mail: zshi@jlu.edu.cn

[c]Department of Chemistry, Oregon State University, 2100 SW Monroe Ave. Corvallis, OR, USA, 97331, USA.

[d]College of Resource and Environmental Science, Jilin Agricultural University, Changchun 130118, China.

Methods

All reagents were purchased in high purity grade from Fisher Scientific, Sigma-Aldrich, Alfa and used without further purification. PAF-1,[1] LDHs,[2] ITC-4,[3] ZIF-8,[4] UiO-66,[5] MIL-101-Cr,[6] PVBTAH~ZIF-8,[7] SLUG-21[8] were synthesized according to the procedures in the references. Solvents were purified according to standard methods and stored in the presence of molecular sieves. The active site based ion exchange kinetics and capacity for stable ion exchange materials of PAF-1-$CH_2N^+(CH_3)_3Cl^-$ and PAF-1-$CH_2N^+(CH_3)_3Cl^-$, Amberlyst-A26, and LDHs were evaluated by normalizing the results from ion exchange tests. The content of nitrogen in Amberlyst-A26 was derived by elemental analysis: C: 64.83%, H: 8.437%, N: 4.50%. The content of active site for LDHs $[Mg_3Al(OH)_8]NO_3.2H_2O$ was calculated from its formula.

<u>Synthesis of PAF-1</u>

Tetrakis(4-bromophenyl)methane (509 mg, 0.8 mmol) was added to a solution of 2,2'-bipyridyl (565 mg, 3.65 mmol), bis(1,5-cyclooctadiene)nickel(0) (1.0 g, 3.65 mmol), and 1,5-cyclooctadiene (0.45 mL, 3.65 mmol) in anhydrous DMF/THF (60 mL/90 mL), and the mixture was stirred overnight at room temperature under nitrogen atmosphere. After the reaction, 6 M HCl (60 mL) was added slowly, and the resulting mixture was stirred for 12 h. The precipitate was collected by filtration, then washed with methanol and water, and dried at 150 °C for 24 h under vacuum (80 mbar) to produce PAF-1 as a white powder, yield: 80%.

<u>The model experiment of extracting MnO_4^- from aqueous solutions</u>

A 50 mL aqueous solution of $KMnO_4$ (10 mg) was added to a 125 mL vial, which was followed by the addition of 25.0 mg samples to form a slurry. During the stirring period, the mixture was filtered at intervals through a 0.45 micron membrane filter for all samples, then the filtrates were analyzed using UV-Vis to determine the concentration of MnO_4^- ions.

<u>Ion exchanged capacity test</u>

The ion-exchange capacity of PAF-1-$CH_2N^+(CH_3)_3Cl^-$ was determined on dry weight basis by a traditional titration method.[9] Typically, 0.5 g PAF-1-$CH_2N^+(CH_3)_3Cl^-$ was

suspended in 5 mL DI water. To this suspension, 0.5 g $NaNO_3$ was added and kept stirring for 5 hours. Then, the mixture was titrated by 0.01 M $AgNO_3$ solution with potassium chromate as indicator.

Exchange kinetics of $Au(CN)_2^-$

A 500 mL aqueous of $KAu(CN)_2$ (15 ppm) was added to a Erlenmeyer flask. Then 1.0 g PAF-1-$CH_2N^+(CH_3)_3OH^-$ sample or Amberlyst-A26 was added to form a slurry. During the stirring period, the mixture was filtered at intervals through a 0.45 micron membrane filter for all samples, then the filtrates were analyzed using ICP-MS to quantify the gold cyanide amount. To obtain the k_2 value, the experimental data was fitted with the pseudo-second-order kinetic model using the following equation:

$$\frac{t}{q_t} = \frac{1}{k_2 q_e^2} + \frac{t}{q_e} \quad \text{(S1)}$$

where k_2 (g mg^{-1} min^{-1}) is the rate constant of pseudo-second-order adsorption, q_t (mg g^{-1}) is the amount of Au(I) adsorbed at time t (min), and q_e (mg g^{-1}) is the amount of Au(I) adsorbed at equilibrium. The distribution coefficient K_d is defined as: [10]

$$K_\mathrm{d} = \frac{(C_\mathrm{i} - C_\mathrm{f})}{C_\mathrm{f}} \times \frac{V}{m} \quad \text{(S2)}$$

where C_i is the initial metal ion concentration, C_f is the final equilibrium metal ion concentration, V is the volume of the treated solution [mL], and m is the mass of sorbent used [g].

Exchange isotherms of $Au(CN)_2^-$

PAF-1-$CH_2N^+(CH_3)_3OH^-$ and Amberlyst-A26 (100.0 mg) were added to each Erlenmeyer flask containing $KAu(CN)_2$ solution (50 mL) with different concentrations. The mixtures were stirred at room temperature for 3 h, and then were filtered separately through a 0.45 micron membrane filter, and the filtrates were analyzed by using ICP-MS to determine the remaining Au(I) content. Langmuir isotherm equation has been used to analyze the experimental data. The maximum working capacity were calculated based on the following equation: [11]

$$\frac{C_e}{q_e} = \frac{1}{\mathrm{Q}_0 b} + \frac{C_e}{\mathrm{Q}_0} \quad \text{(S3)}$$

where C_e and q_e are Au(I) equilibrium concentrations in the aqueous and the Au(I) uptake

amount for 1 g sample, respectively; Q_0 is maximum uptake capacity of the adsorbent, and b is the Langmuir adsorption constants related to the free energy of adsorption. The fitting data are shown in Fig. S8 and S9.

Ionic conductivity (S/cm) was calculated using the formula below:

$$\sigma = L/(AR) \qquad \text{(S4)}$$

where L is the pellet thickness while A is the pellet area in contact with the stainless- steel electrodes. R is the complex impedance obtained from Nyquist plot. The detail parameters: $Au(CN)^-$@PAF-1-$CH_2N^+(CH_3)_3OH^-$ (L = 0.16 cm; A = 1.22 cm^2; R: 0.406×10^6) and $Au(CN)^-$@Amberlyst-A26 (L = 0.13 cm; A = 1.22 cm^2; R: 0.880×10^6). Grind resin Amberlyst-A26 and PAF-1-$CH_2N^+(CH_3)_3OH^-$ were degas at 60 ºC under vacuum for 3 h before ion-exchange experiment.

Selective capture of anionic guests via size-exclusion effect

The experiments were performed by immersing PAF-1-$CH_2N^+(CH_3)_3Cl^-$ (20 mg) in the 20 mL dye OG/MB (0.083 mM:0.083 mM) aqueous. UV–vis absorbance measurements were performed periodically for the filtered solution, which was poured back to the original system after each measurement to keep the total amount of sample constant. The peak of MB were normalized as 100% in order to calculate the OG content in remaining solution. The Amberlyst-A26, LDHs and ITC-4 were tested with similar procedure.

Reversible ion exchange experiment

Gold cyanide ion exchange for cycle

A 50 mL aqueous of $KAu(CN)_2$ (450 ppm) was added to a Erlenmeyer flask. Then 100 mg PAF-1-$CH_2N^+(CH_3)_3OH^-$ was added to form a slurry. After the 1 h, the mixture was filtered at intervals through a 0.45 micron membrane filter, then the filtrates were analyzed using ICP-MS to quantify the gold cyanide amount. Gold cyanide load PAF-1-$CH_2N^+(CH_3)_3OH^-$ (Au content: 1.03 mmol g^{-1}) was soaked in 100 mL stripping solutions include 1 M NaOH ethanolic solution (water : ethanol, 1:1 v/v) and the gold cyanide ion elution efficiency is calculated according to Equation and quantified by ICP-MS as a function of time.

$$\text{Elution efficiency (\%)} = \frac{\text{gold striping amount}}{\text{gold extraction amount}} \times 100 \qquad \text{(S5)}$$

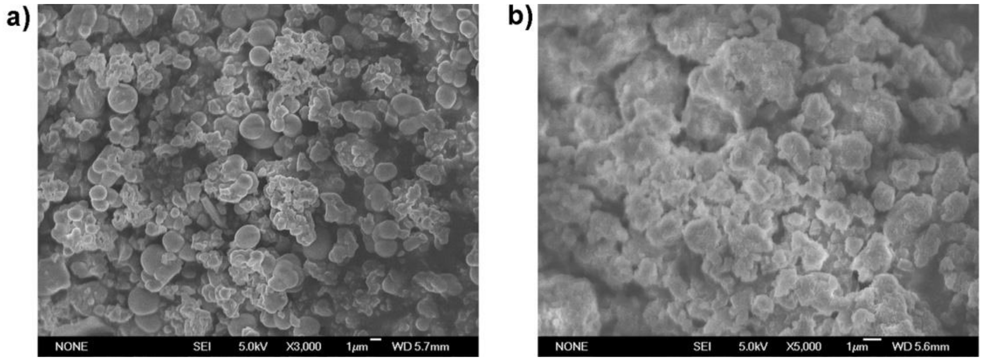

Fig. S1 SEM image of samples. (a) PAF-1-$CH_2N^+(CH_3)_3OH^-$; (b)Amberlyst-A26 ion-exchange resin.

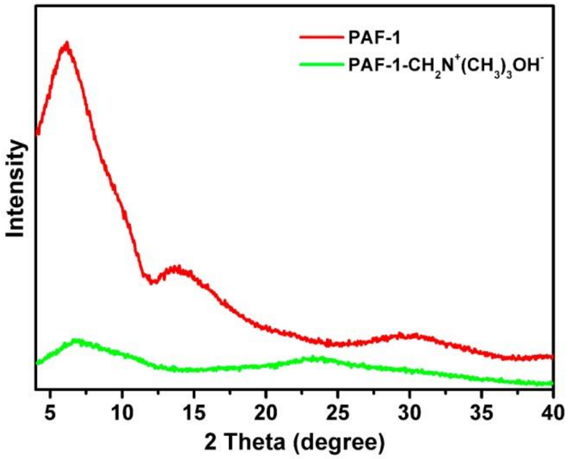

Fig. S2 PXRD patterns of PAF-1 and PAF-1-$CH_2N^+(CH_3)_3OH^-$.

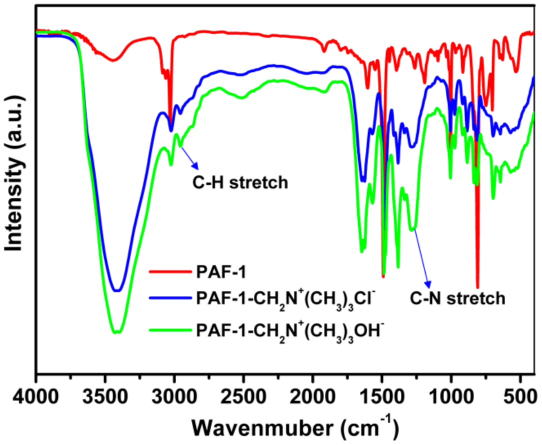

Fig. S3 FT-IR spectra of PAF-1, PAF-1-$CH_2N^+(CH_3)_3Cl^-$ and PAF-1-$CH_2N^+(CH_3)_3OH^-$.

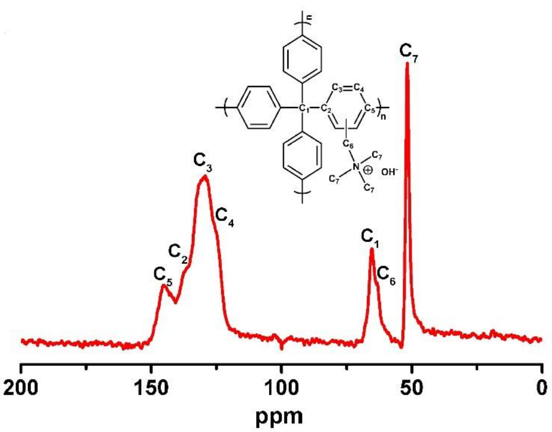

Fig. S4 ^{13}C NMR spectra of PAF-1-$CH_2N^+(CH_3)_3OH^-$.

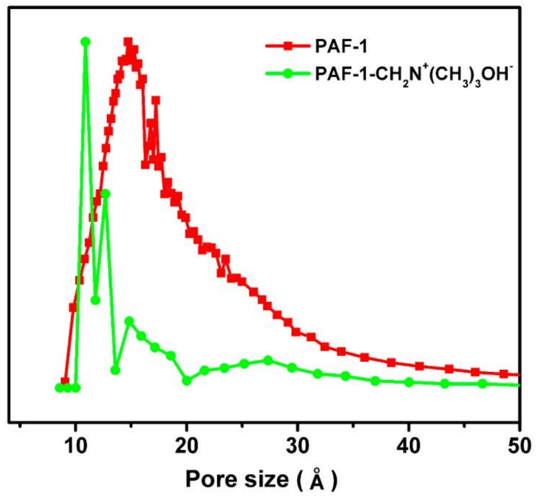

Fig. S5 Pore size distributions of PAF-1 and PAF-1-$CH_2N^+(CH_3)_3OH^-$.

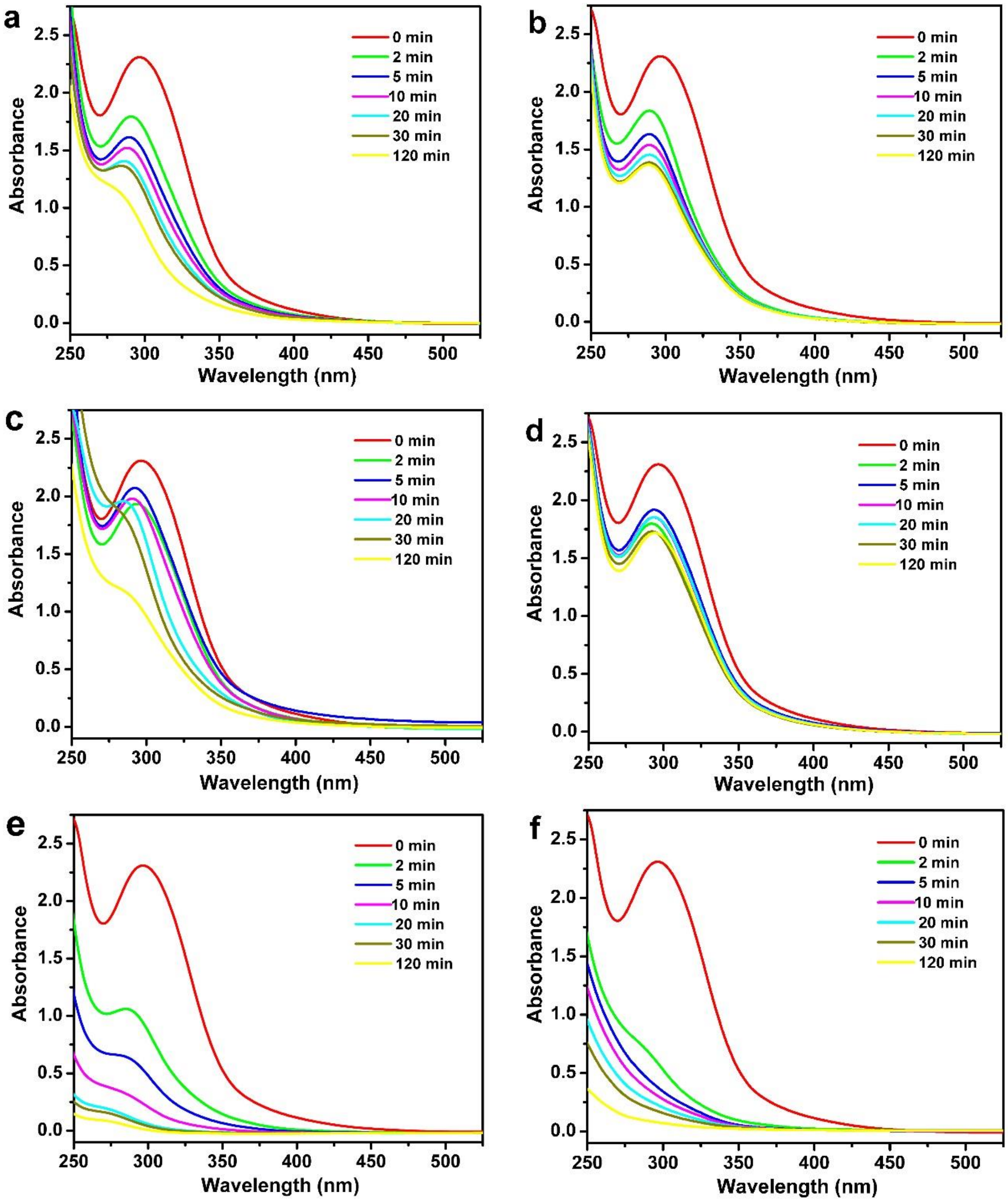

Fig. S6 UV–vis spectra of $AuCl_4^-$ aqueous solutions in the presence of (a) LDHs, (b) ITC-4, (c) ZIF-8, (d) PAF-1, (e) Amberlyst-A26 and (f) PVBTAH~ZIF-8 monitored with time.

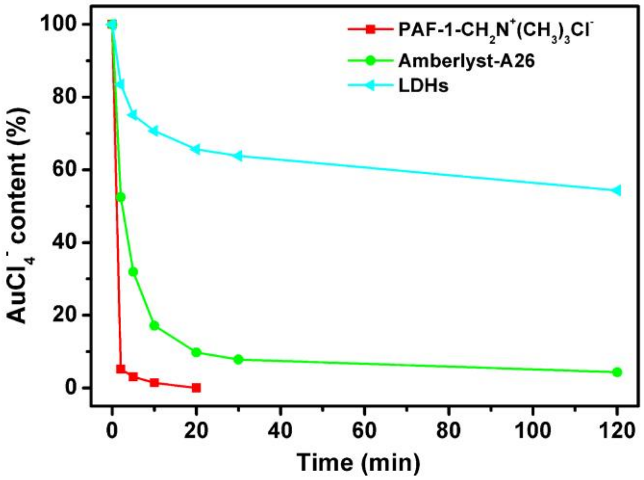

Fig. S7 Comparison of the ion exchange performances for PAF-1-$CH_2N^+(CH_3)_3Cl^-$ and other ion exchange materials in extracting $AuCl_4^-$ based on the same mol of active exchanged sites.

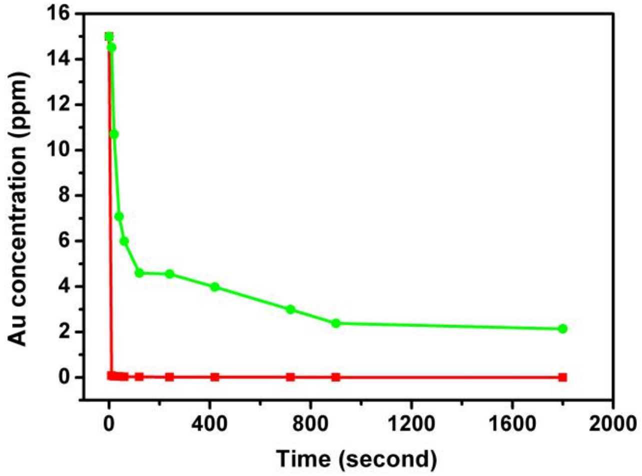

Fig. S8 $Au(CN)_2^-$ exchange kinetics of PAF-1-$CH_2N^+(CH_3)_3OH^-$ (red) and Amberlyst-A26 (green) with Au(I) initial concentration of 15 ppm in $KAu(CN)_2$ solution based on the same mol of active exchanged sites.

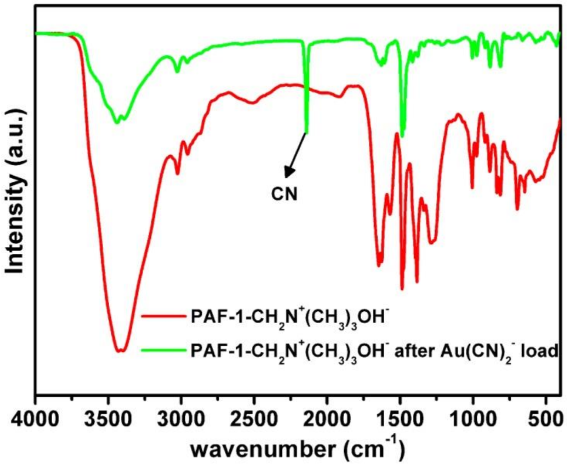

Fig. S9 FT-IR spectra of PAF-1-$CH_2N^+(CH_3)_3OH^-$ and $Au(CN)_2^-$ loaded PAF-1-$CH_2N^+(CH_3)_3OH^-$.

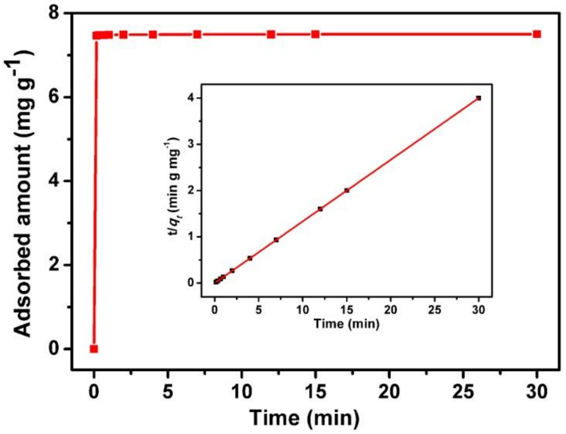

Fig. S10 Adsorption curve of $Au(CN)_2^-$ versus contact time in aqueous solution by using the PAF-1-$CH_2N^+(CH_3)_3OH^-$. Inset shows the pseudo-second-order kinetic plot for the adsorption.

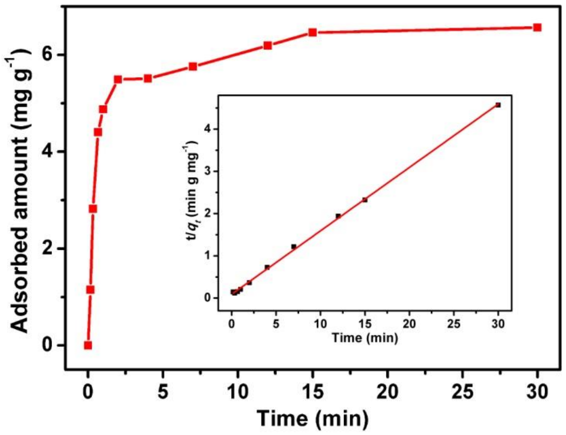

Fig. S11 Adsorption curve of Au(I) versus contact time in aqueous solution by using the Amberlyst-A26. Inset shows the pseudo-second-order kinetic plot for the adsorption.

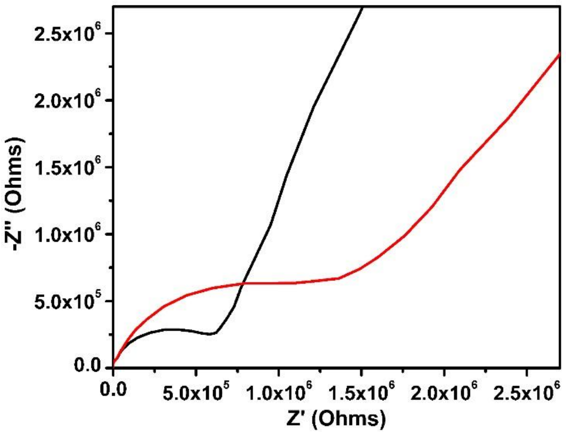

Fig. S12 Nyquist plots of $Au(CN)_2^-$@ PAF-1-$CH_2N^+(CH_3)_3OH^-$ (black) and $Au(CN)_2^-$@Amberlyst-A26 (red).

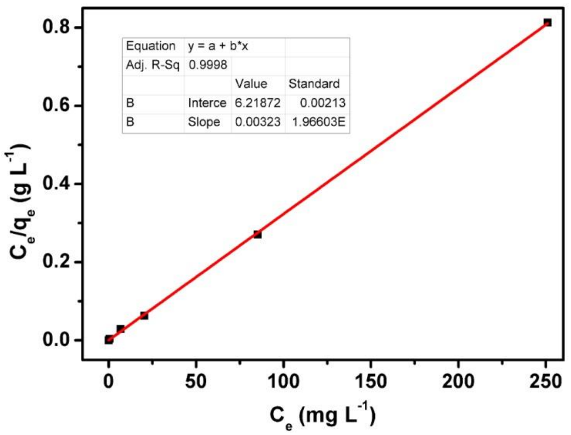

Fig. S13 Fitting of adsorption isotherm on the basis of Langmuir model for Au(I) on PAF-1-$CH_2N^+(CH_3)_3OH^-$.

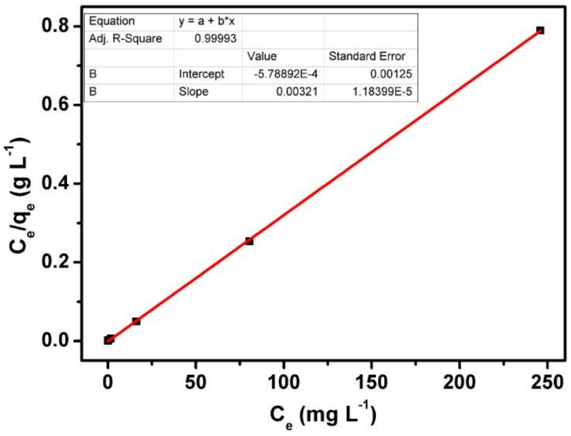

Fig. S14 Fitting of adsorption isotherm on the basis of Langmuir model for Au(I) on Amberlyst-A26.

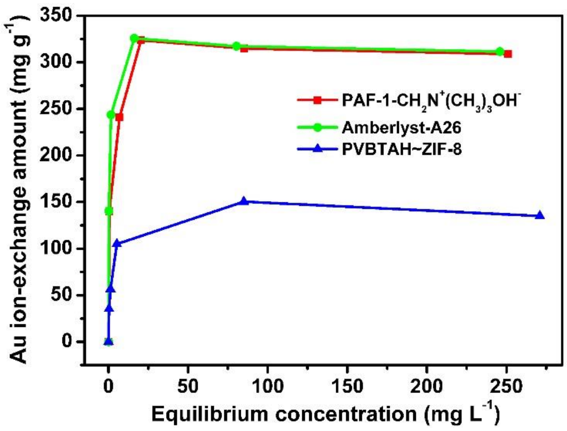

Fig. S15 Comparison of Au(I) adsorption isotherms based on dry weight for $PAF\text{-}1\text{-}CH_2N^+(CH_3)_3OH^-$, Amberlyst-A26 and PVBTAH~ZIF-8.

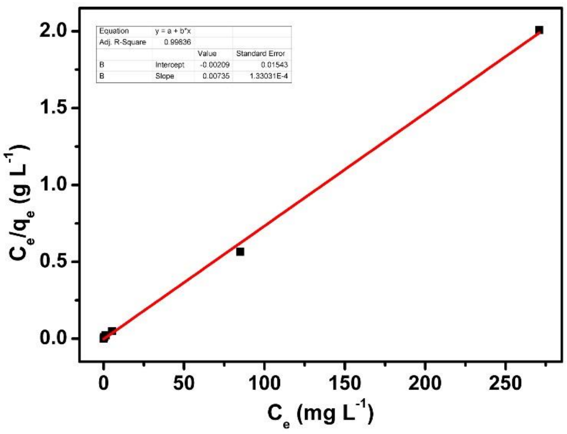

Fig. S16 Fitting of adsorption isotherm on the basis of Langmuir model for Au(I) on PVBTAH~ZIF-8.

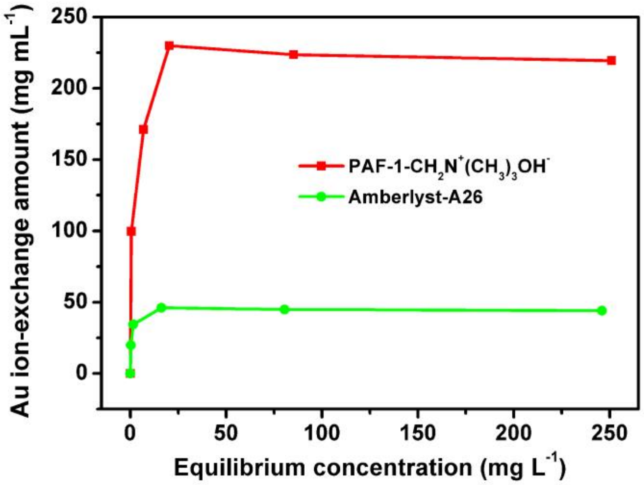

Fig. S17 Volumetric Au(I) adsorption isotherms for PAF-1-$CH_2N^+(CH_3)_3OH^-$, Amberlyst-A26 based on the same mol of active exchange sites.

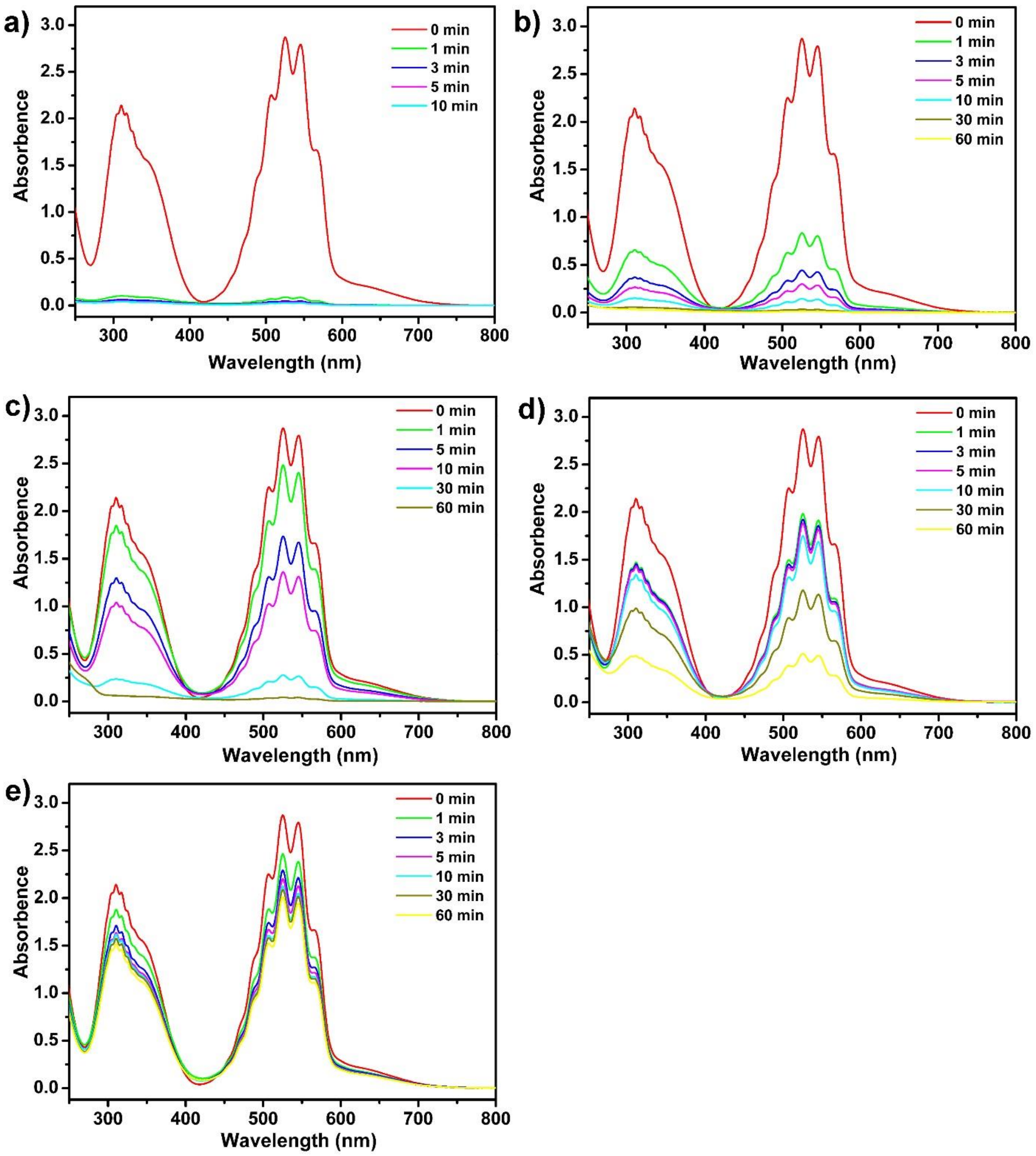

Fig. S18 UV–vis spectra of MnO_4^- aqueous solutions in the presence of (a) PAF-1-$CH_2N^+(CH_3)_3OH^-$, (b) Amberlyst-A26, (c) SLUG-21, (d) PVBTAH~ZIF-8 and (e) LDHs monitored with time.

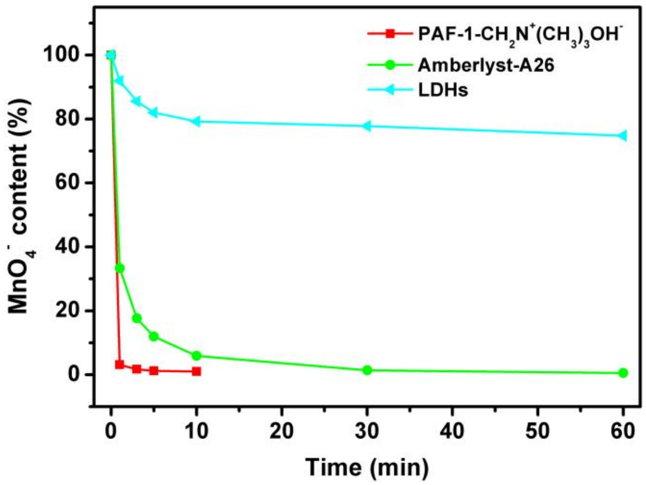

Fig. S19 Comparison of the ion exchange performances of PAF-1-$CH_2N^+(CH_3)_3OH^-$ and other ion exchange materials in removing model MnO_4^- ions based on the same mol of active exchanged sites.

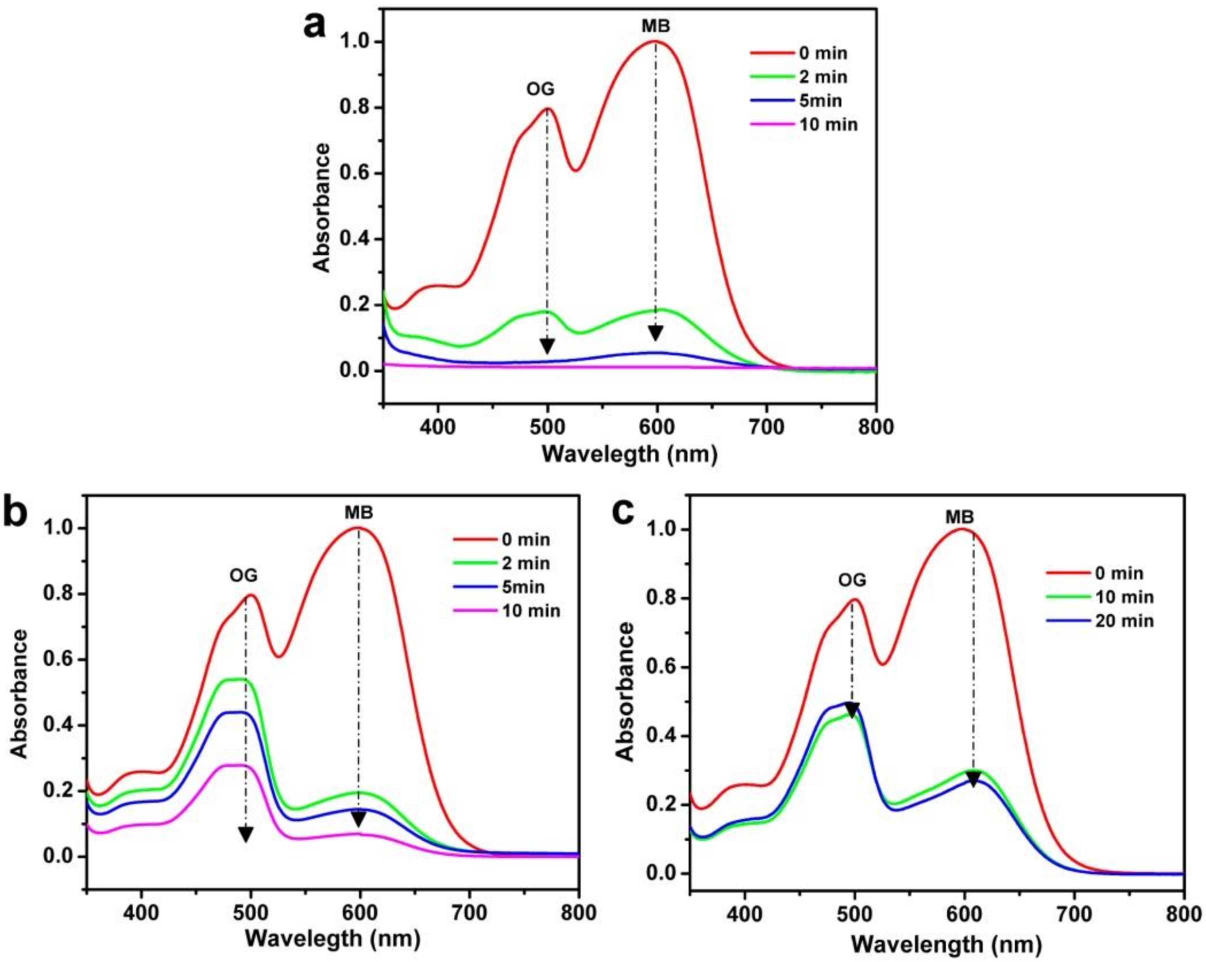

Fig. S20 UV–vis spectra of OG/MB aqueous solutions in the presence of (a) Amberlyst-A26, (b) LDHs, and (c) ITC-4.

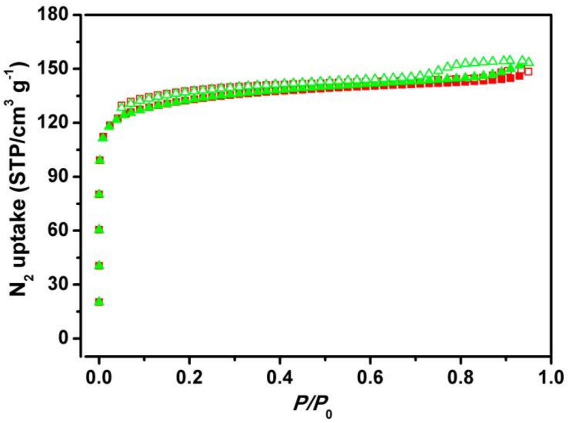

Fig. S21 N_2 sorption isotherms of PAF-1-$CH_2N^+(CH_3)_3OH^-$ (red: as-synthesized; green: after acid and base treatments).

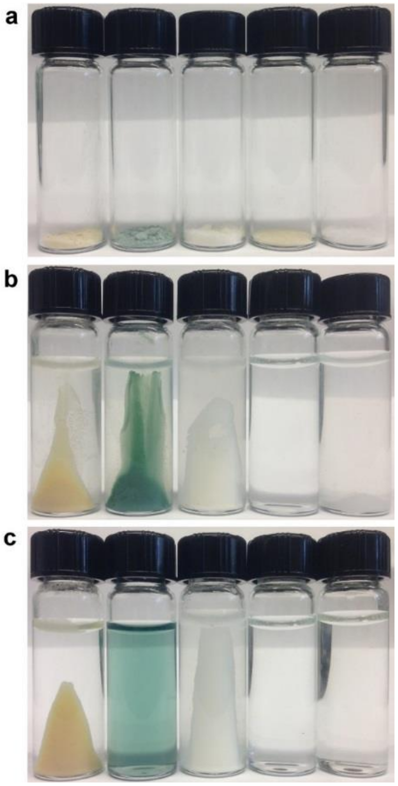

Fig. S22 The photos for different samples after acid and base treatment. (a) The samples before addition of HCl and NaOH; (b) The samples after the treatment of 1 M HCl; (c) The samples after the treatment of 1 M NaOH. The samples from left to right are PAF-1-$CH_2N^+(CH_3)_3OH^-$, MIL-101-Cr, UiO-66, ZIF-8 and MCM-41 respectively. From Fig. S18b, we can see that the ZIF-8 are not stability under the acid conditions. After the treatment of 1 M NaOH (see Fig. S18c), MIL-101-Cr and MCM-41 lost their solid. Only UiO-66 remain some solid in the solution.

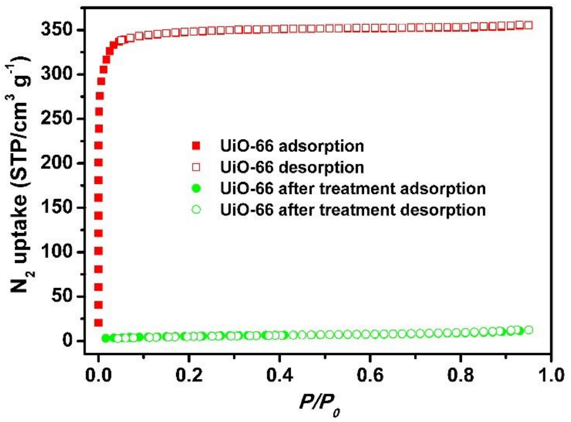

Fig. S23 The N_2 sorption of UiO-66 before and after 1 M HCl and 1 M NaOH treatments. The result indicates the instability of UiO-66 after acid and base treatments.

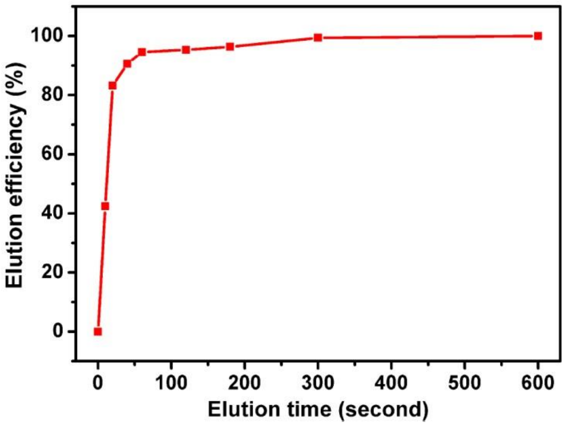

Fig. S24 Gold cyanide ion elution efficiency from the gold cyanide loaded PAF-1-$CH_2N^+(CH_3)_3OH^-$.

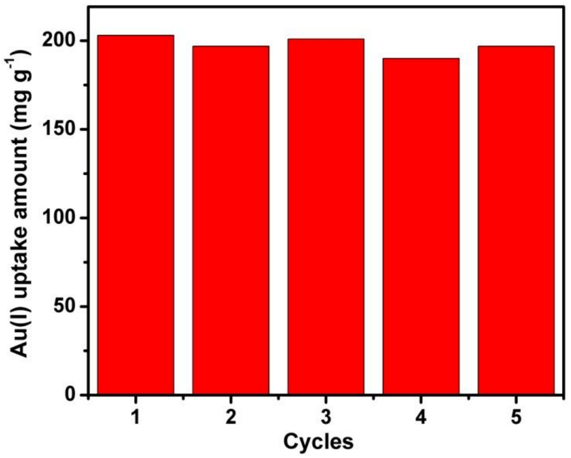

Fig. S25 Recyclability of PAF-1-$CH_2N^+(CH_3)_3OH^-$ for gold cyanide ion extraction.

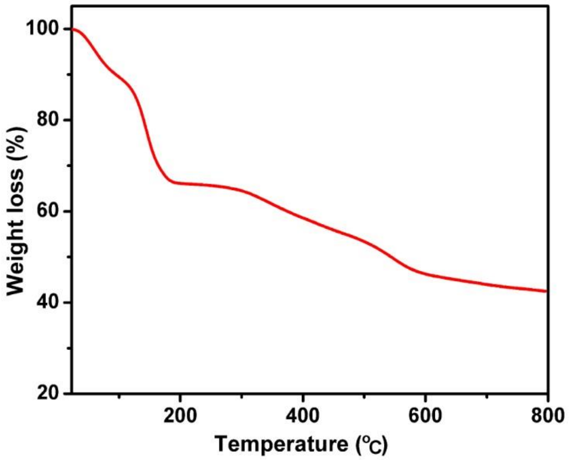

Fig. S26 TGA plots PAF-1-$CH_2N^+(CH_3)_3OH^-$.

Table S1 The comparison of properties between two ion exchanged materials including PAF-1-$CH_2N^+(CH_3)_3OH^-$ and Amberlyst-A26 ion-exchange resin.

sample	S_{BET} (m^2 g^{-1})	Density (g cm^{-1})	Pore size (nm)	Ion-exchange capacity By wt (meq g^{-1})	By vol (meq ml^{-1})	k_2 (g mg^{-1} min^{-1})[a]	K_d (mL g^{-1})[b]	Working capacity[c] By wt (meq g^{-1})	By vol (meq mL^1)
PAF-1-$CH_2N^+(CH_3)_3OH^-$	**505**	**0.71**[d]	**1.1**	**3.4**[e]	**2.4**[f]	**50.4**	**4.6×10^6**	**1.6**	**1.1**
Amberlyst -A26	**30**[g]	**0.64**[g]	**irregular macropore**	**4.4**[h]	**0.8**[i]	**0.25**	**3.5×10^3**	**1.6**	**0.29**[j]

[a)]k_2 is the rate constant of pseudo-second-order adsorption for $Au(CN)_2^-$. [b)]K_d is the distribution coefficient which is defined as the amount of exchanged ion (mg) on 1 g of ion-exchange material divided by the ion concentration (mg mL^{-1}) remaining in the solution. [c)]The maximum working capacity for $Au(CN)_2^-$ is calculated based on Langmuir model. [d)]The density is measured by compressed pellet of sample. [e)]The value is measured by titration method. [f)]Converted from measure density as noted in (*d*). [g)]The value is from ref. 7. [h)]Data from Sigma-Aldrich for dry resin in Cl form. [i)]Data from Sigma-Aldrich for a wet resin. [j)]Converted based on the swelling coefficient of Amberlyst-A26 for dry sample and wet sample.

References:

1 B. Li, Y. Zhang, R. Krishna, K. Yao, Y. Han, Z. Wu, D. Ma, Z. Shi, T. Pham, B. Space, J. Liu, P. K. Thallapally, J. Liu, M. Chrzanowski, S. Ma, *J. Am. Chem. Soc.* 2014, **136**, 8654.

2 M. Meyn, K. Beneke, G. Lagaly, *Inorg. Chem.* 1990, **29**, 5201.

3 X. Zhao, X. Bu, T. Wu, S.-T. Zheng, L. Wang, P. Feng, *Nat. Commun.* 2013, **4**, 2344.

4 K. S. Park, Z. Ni, A. P. Côté, J. Y. Choi, R. Huang, F. J. Uribe-Romo, H. K. Chae, M. O'Keeffe, O. M. Yaghi, *Proc. Natl. Acad. Sci.* 2006, **103**, 1018.

5 J. H. Cavka, S. Jakobsen, U. Olsbye, N. Guillou, C. Lamberti, S. Bordiga, K. P. Lillerud, *J. Am. Chem. Soc.* 2008, **130**, 13850.

6 G. Férey, C. Mellot-Draznieks, C. Serre, F. Millange, J. Dutour, S. Surblé and I. Margiolaki, *Science* 2005, **309**, 2040.

7 L. Gao, C.-Y. V. Li, K.-Y. Chan, Z.-N. Chen, *J. Am. Chem. Soc.* 2014, **136**, 7209.

8 H. Fei, M. R. Bresler, S. R. J. Oliver, *J. Am. Chem. Soc.* 2011, **133**, 11110.

9 C. E. Harland, Ion-exchange: Theory and Practicle, 2nd ed., Royal Society of Chemistry: London,

(1994).
10 X. Feng, G. E. Fryxell, L.-Q. Wang, A. Y. Kim, J. Liu, K. M. Kemner, *Science* 1997, **276**, 923.
11 Y. Zhang, Z.-A. Qiao, Y. Li, Y. Liu, Q. Huo, *J. Mater. Chem.* 2011, **21**, 17283.
